# Clinical Application of Epilepsy Genetics in Africa: Is Now the Time?

**DOI:** 10.3389/fneur.2018.00276

**Published:** 2018-05-02

**Authors:** Alina I. Esterhuizen, Gemma L. Carvill, Rajkumar S. Ramesar, Symon M. Kariuki, Charles R. Newton, Annapurna Poduri, Jo M. Wilmshurst

**Affiliations:** ^1^Division of Human Genetics, Department of Pathology, Institute of Infectious Diseases and Molecular Medicine, University of Cape Town, Cape Town, South Africa; ^2^National Health Laboratory Service, Groote Schuur Hospital, Cape Town, South Africa; ^3^Ken and Ruth Davee Department of Neurology, Feinberg School of Medicine, Northwestern University, Chicago, IL, United States; ^4^KEMRI-Wellcome Trust Research Programme, Centre for Geographic Medicine Research-Coast, Kilifi, Kenya; ^5^Department of Psychiatry, University of Oxford, Oxford, United Kingdom; ^6^Department of Neurology, Harvard Medical School, Boston, MA, United States; ^7^Department of Neurology, Epilepsy Genetics Program, Boston Children’s Hospital, Boston, MA, United States; ^8^School of Child and Adolescent Health, University of Cape Town, Cape Town, South Africa; ^9^Paediatric Neurology and Neurophysiology, Red Cross War Memorial Children’s Hospital, Cape Town, South Africa

**Keywords:** low- to middle-income countries, genetic testing, seizures, sub-Saharan Africa, genetic epilepsy, early-life epilepsy

## Abstract

Over 80% of people with epilepsy live in low- to middle-income countries where epilepsy is often undiagnosed and untreated due to limited resources and poor infrastructure. In Africa, the burden of epilepsy is exacerbated by increased risk factors such as central nervous system infections, perinatal insults, and traumatic brain injury. Despite the high incidence of these etiologies, the cause of epilepsy in over 60% of African children is unknown, suggesting a possible genetic origin. Large-scale genetic and genomic research in Europe and North America has revealed new genes and variants underlying disease in a range of epilepsy phenotypes. The relevance of this knowledge to patient care is especially evident among infants with early-onset epilepsies, where early genetic testing can confirm the diagnosis and direct treatment, potentially improving prognosis and quality of life. In Africa, however, genetic epilepsies are among the most under-investigated neurological disorders, and little knowledge currently exists on the genetics of epilepsy among African patients. The increased diversity on the continent may yield unique, important epilepsy-associated genotypes, currently absent from the North American or European diagnostic testing protocols. In this review, we propose that there is strong justification for developing the capacity to offer genetic testing for children with epilepsy in Africa, informed mostly by the existing counseling and interventional needs. Initial simple protocols involving well-recognized epilepsy genes will not only help patients but will give rise to further clinically relevant research, thus increasing knowledge and capacity.

## Introduction

**Figure 1:**
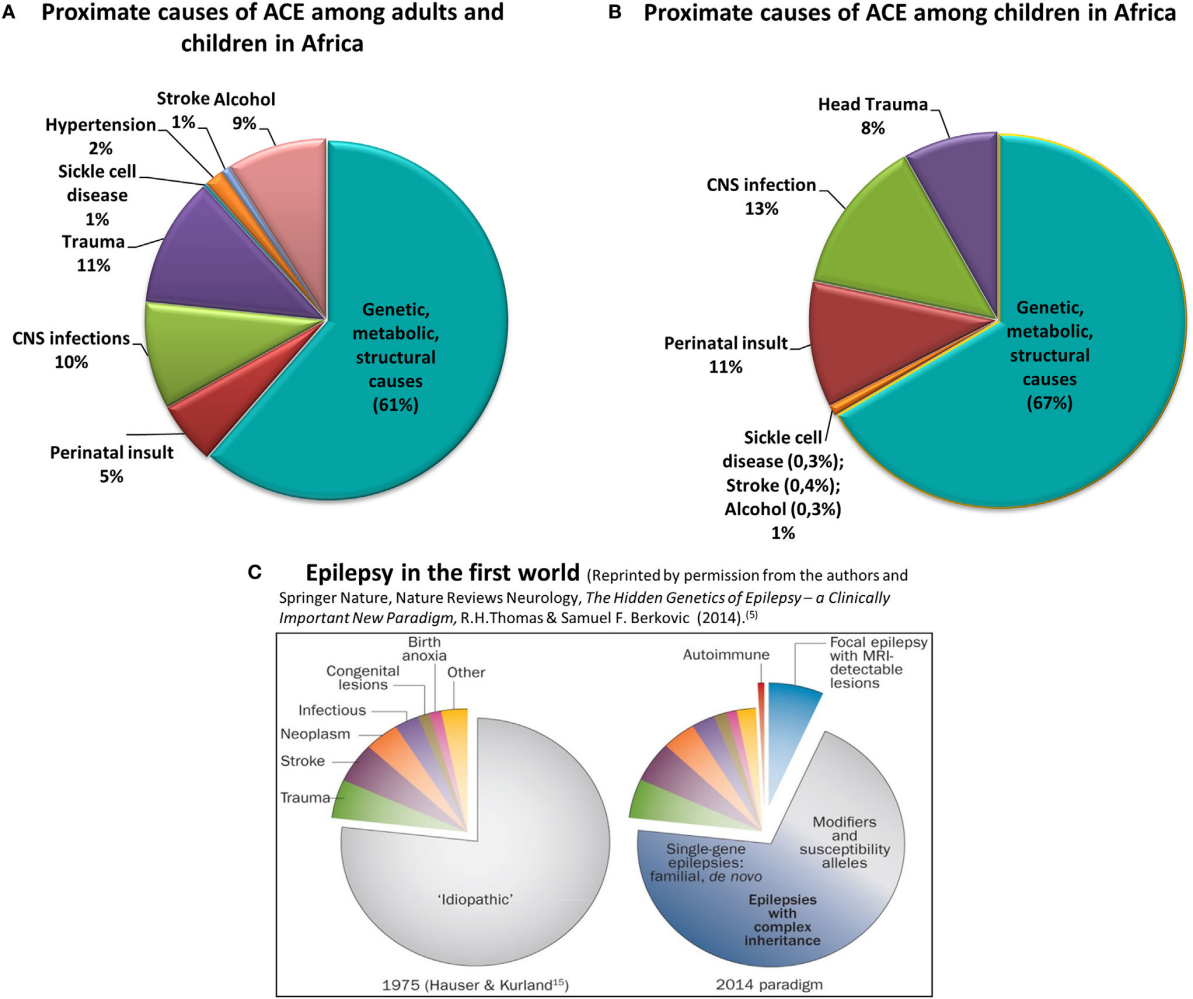
Proximate causes of active convulsive epilepsy (ACE) in Africa according to currently available knowledge. Charts **(A,B)** depict the distribution of proximate causes of ACE among all ages **(A)** and among children in Africa **(B)** [data obtained from Kariuki et al. (3) and personal communication with coauthors]. Chart **(C)** reflects the causes of all epilepsies in the first-world environment (5). Charts **(A–C)** clearly show the significantly higher burden of epilepsy due to central nervous system infections, trauma, and perinatal insult in Africa, particularly among children. Also emphasized is the absence of reliable knowledge on the burden of the genetic, metabolic, and structural causes of epilepsy in Africa.

Epilepsy affects approximately 70 million people globally. Of these, over 80% live in low- to middle-income countries (LMICs) (1) where epilepsy is under-diagnosed and often untreated (2). The underlying reasons range from poorly resourced healthcare systems to the social stigma of epilepsy and reluctance to seek treatment. The high prevalence of epilepsy, particularly in sub-Saharan Africa (SSA) coexists with increased risk factors, especially central nervous system infections, perinatal insults, and traumatic brain injury (3). Epilepsy due to genetic, immune, metabolic, or structural causes is rarely recognized, and its burden is virtually unknown (Figure 1) (3).

The causal role of genetic variants in epilepsy is increasingly recognized. Over the past two decades, large-scale studies empowered by genomic technologies have shown that many epilepsies previously classified as “idiopathic” have a genetic basis (4, 5) (Figure 1). Effective investigation of genetically and phenotypically complex disorders such as epilepsy requires laboratory protocols incorporating next-generation sequencing (NGS) and chromosomal microarrays; now routinely employed in the diagnostic centers of high income countries (HICs). In Africa, however, resource allocation for genetic services is not prioritized, thus the necessary skills and equipment are lacking. Genetic epilepsy research is practically absent, resulting in few insights into the architecture of the disease in African populations.

In this review, we examine the historical and current demographics of epilepsy and the medical diagnostic infrastructure in Africa. Within the context of global genetic research and its impact on personalized medicine, we argue that the time for epilepsy genetics in Africa is now and propose tangible actions to improve access to genomic technologies and diagnostic testing.

## Disease Burden and Management of Epilepsy in Africa

The Global Burden of Diseases, Injuries, and Risk Factors Study 2010, reported the burden of untreated severe epilepsy second only to HIV infection (6). Epidemiological studies over the past decade show that SSA carries the greatest prevalence and disability burden of epilepsy in the world, with the median prevalence estimated at 14.2 per 1,000 (IQR 8.0–33.2), more than double the prevalence in HICs [5.8 per 1,000 (2.7–12.4)] (3, 7). The true prevalence is likely to be higher, as many cases are not reported and seizures with fewer motor manifestations often go unrecognized (7, 8).

Almost 60% of people with epilepsy in SSA do not receive medication and only about 33% of those who do are appropriately managed (7). The likely reflection of this is the substantial risk of premature mortality in people with epilepsy in Africa, reported at rates ranging from 22.2 to 45.1 per 1,000 (9). In addition to causes of death directly attributable to epilepsy, e.g., status epilepticus, many die from seizure-related drowning, head injury or burns (9). Epilepsy also carries with it a significant “social disability” aspect, which is not reflected by the disability weight estimates. Reduced marriage prospects, less access to education, and lower employment opportunities all place an additional burden on the individual and the family (7, 10).

The “treatment gap” for epilepsy in Africa, defined as a percentage of people living with active untreated epilepsy is 47% in urban regions, compared with 73% in the rural areas, where prognosis and outcomes are poor (11). Treatment guidelines are usually created in well-resourced environments and require major adaptation to fit the African context (11). The recommended diagnostic tools and newer generation antiepileptic drugs (AEDs) are available only in the major tertiary centers of the state sector or in private practice (12). Typically, the correct diagnosis and treatment of early-life epilepsies in African children is achieved months or years after initial presentation and many die undiagnosed. The resulting financial burden placed on the healthcare system in these situations could be alleviated by early genetic diagnoses and timely intervention (13).

In South Africa, non-communicable diseases (NCDs) gained statistical prominence in the early 1990s, only to recede under the burden of the HIV/AIDS and TB pandemics (14). In 2015, however, the infant mortality rate dropped below 40/1,000 live births, signaling the need for resource allocation toward better service provision for NCDs (15). Recognition of the immediate and long-term value of genetic services is an imperative part of this transition.

## Epilepsy as a Genetic Disease

The major finding of epilepsy research in recent years is the high prevalence of *de novo* pathogenic variants, particularly well noted in developmental and epileptic encephalopathies (DEEs) (16–19). DEEs are characterized by pharmacoresistant seizures, severe electroencephalography (EEG) abnormalities, and developmental delay (DD)/regression/intellectual disability. Approximately 40% of seizures with onset in the first 3 years of life will progress to DEE, and a substantial number of these are associated with variants in known epilepsy genes (20). While the majority of EE patients carry *de novo* pathogenic changes, recent studies suggest that parental somatic mosaicism is present in up to 10% of the cases (21). This has important implications for genetic counseling and family planning, as the risk of recurrence in these families may be as high as 50% (21).

Although clinical and genetic heterogeneity is the hallmark of genetic epilepsy, certain genes and variants co-exist with characteristic clinical features. For instance, pathogenic *SCN1A* variants are identified in 80% of patients with Dravet syndrome (22). Movement disorders and head stereotypies are often seen with *STXBP1* variants (23) and clustered focal seizures with affective symptoms are seen in females with *PCDH19* variants (24). Awareness of such features is important in implementing cost-effective testing with a high yield of informative results. Furthermore, disease-causing genes in the severe forms of epilepsy are also implicated in a broader spectrum of epilepsy and associated neurodevelopmental disorders (25). Therefore, testing and therapeutic protocols targeting rare genetic epilepsies may also find application among the more common phenotypes (26).

## Genetic Testing for Epilepsy

The value of genetic testing in epilepsy has been debated and depends on the phenotype and reason for testing. Presently, testing for early-onset epilepsies appears to yield the most informative results, as recently emphasized in a report by Berg and colleagues. In a group of 327 children with seizure onset before the third year of life who underwent some form of genetic testing, pathogenic variants were identified in 132 (40.4%) of the cases (27). While clinical whole-exome sequencing is gaining popularity as a first tier assay for genetically heterogeneous disorders, the targeted approach is mostly still favored in the clinical setting. NGS panels have increased specificity, greater depth of coverage (better sensitivity), less exonic dropout, and fewer issues relating to incidental findings (13, 28). The main disadvantage is missing a pathogenic change in a gene not included in the panel. Chromosomal microarray analysis for genomic copy number variants (CNVs) is indicated in children with seizures accompanying DD/intellectual disability, as there is evidence that up to 10% of these patients have disease-associated CNVs (29).

Genomic technologies are expensive to establish and maintain, and require technical and bioinformatic expertise. In South Africa, the established infrastructure for genetic services includes laboratories and clinical services. Regular specialist clinics and outreach initiatives strive to increase awareness and deliver services both locally and to the neighboring, more resource-limited SSA countries, highlighting the need to build capacity across the continent. Despite the challenges of diagnosis and treatment and even in absence of population-specific data, there is sufficient justification to support availability of early screening protocols for specific epilepsy phenotypes.

Neuroimaging, ideally magnetic resonance imaging (MRI), and EEG remain essential investigations in the diagnosis of structural brain pathology. However, for many generalized epilepsies, MRI and EEG findings lack the specificity and consistency of an informative genetic test result. Most DEEs are genetic in origin, with an increasing number of clinical markers linked to specific genetic etiologies (27). Access to neuroimaging and EEG in some LMICs is very limited, while DNA studies on saliva may become far more accessible at a similar cost. Therefore, resource allocation for genetic testing versus MRI should be prioritized on the basis of the clinical semiology, for early and targeted care (30).

The largely *de novo* etiology of DEE shown in the HICs, should not be vastly different in Africa and while new, “African” epilepsy genes may emerge with future research, the DEE panels currently used in the HICs should also benefit African patients. Genetic testing may sometimes present a more direct and cheaper diagnostic tool than the traditional options, prevent the use of potentially seizure-exacerbating therapies (e.g., carbamazepine for *SCN1A*-associated DS) and further invasive and expensive investigations. These aspects are equally important to patients and families all over the world, and the benefits carry far-reaching health, psychological and socioeconomic consequences.

## Epilepsy Research in Africa

The existing reports of neurogenetic research in Africa have shown that the genetic underpinnings of certain neurological phenotypes segregate almost exclusively in African populations. Examples include Huntington disease-like type 2 (31, 32), spinocerebellar ataxia type 7 (SCA7) (33, 34) and *RYR1*-related centronuclear myopathy (35). However, published epilepsy research emanating from the continent focuses on the disease epidemiology, etiology, and management (3, 8, 36–40), and little is known about the genetic causes in African patients. More research is needed to identify the role of presently unknown genetic causes and risk factors for epilepsy in people of African ancestry.

Consanguinity and febrile illness feature strongly as risk factors for epilepsy and genetic disease in general. Both occur frequently in African populations, presenting an excellent focus for research which has not been fully explored. Only a few North African studies of ion channel genes in febrile seizure phenotypes (41, 42) and small cohorts of familial epilepsies in consanguineous families have been published (43–45). For cultural reasons, consanguineous unions are more common in North African countries, compared with the rest of the continent (46). Thus the obvious implications for recessive disease are less relevant to populations in SSA where consanguinity is uncommon, emphasizing the need for population-specific translatable research, genetic counseling, and education. Furthermore, research within the context of the increased genetic diversity in Africa may provide additional insights into the underpinnings of familial epilepsies. The large African sibships and increased twinning in some regions (47, 48) offer a valuable opportunity for genetic studies and a better understanding of brain development, potentially opening the way to the discovery of new therapeutic agents.

The highly variable response to AEDs is complicated by the African diversity and the challenges of managing comorbidities and medications (e.g., malaria, TB, HIV, and schistosomiasis) (49). Many AEDs are substrates for the Cytochrome P450 (CYP) enzymes, which are important determinants of response to most drugs prescribed today. Polymorphisms in the encoding genes are linked to altered levels of activity and adverse drug reactions (50). As an example, individuals carrying the CYP2C9*2 (rs1799853) and CYP2C9*3 [rs1057910(C)] polymorphisms metabolize phenytoin at a markedly slower rate and a higher risk of concentration-dependent neurotoxicity than individuals homozygous for the wild-type allele [CYP2C9*1; rs1057910(A)] (50, 51). However, compared with the European and Asian populations, allele frequencies of these variants among Africans are much lower, negating any real public health applicability in Africa (52, 53). The frequencies of known CYP enzymes show a considerably greater variability across Africa, with a few polymorphisms reported only in African populations. Most research to date focuses on allele frequency distributions of known polymorphisms, and there is a need for discovery of new markers and profiling that is relevant in Africa (54). Adding to this complexity is the widespread use of herbal products, with possible herb–drug interactions which may affect efficacy and toxicity profiles of pharmaceutical drugs (55). Undoubtedly, discovery of new genetic markers of drug response will be of global value, as the ancestral origin of the human population is represented in the African genomes.

While genomic research has been powering ahead in the rest of the world for almost two decades, Africa remains far behind. Insights into the genetic diversity in Africa gained through the Human HapMap, and the 1000 Genomes Project was limited to the Yoruba and Esan (Nigeria), Mende (Sierra Leone), and the Luhya and Masai (Kenya) populations, leaving much of the African continent unexplored (56, 57). If this lag continues, the potential health and economic benefits emanating from genomic science may elude an entire continent (58). Initiatives such as the Human Heredity and Health in Africa project (H3Africa) and the African Genome Variation project (AGV) are designed to urgently address this gap in knowledge and capacity (58, 59), with an emphasis on “Afrocentric” genomic research, biorepositories, and bioinformatic networks (58). This should carry the benefits of improved variant databases, as well as development of preventative and targeted treatments in the age of precision medicine. The increasing burden of NCDs in SSA makes a strong case for more financial and intellectual investment into genetic research in Africa and into translating the outcomes into medical practice.

## Needs and Challenges

Many clinicians in Africa are not trained to recognize a possible genetic epilepsy and focus mainly on prescribing treatment. Signs and symptoms are blurred by the layering effects of untreated seizures and multiple insults of birth trauma, co-infections, nutritional insults, and socioeconomic issues. In SSA, acute symptomatic and febrile seizures are frequently assumed to be due to malaria, limiting the search for other causes. Little consideration is given to the increased subsequent risk for epilepsy, much less the possible genetic basis for this risk (60). Often, a genetic etiology is considered only when a second affected child is born. In the setting where families are already struggling to cope with the complex health care of the first affected child, this can become untenable. Efficient management of epilepsy is particularly important in childhood because of the detrimental effects of uncontrolled seizures on the developing brain (1, 61, 62). The prevailing social stigma of epilepsy often labels these children as infectious, mentally ill or spiritually “possessed” (36, 63). It is important to recognize the potential value of community leaders, elders, and traditional healers (THs), in addressing these issues (THs).

Many Africans, particularly in the rural setting, are inclined to seek treatment from a TH, rather than a “Western-style” medical doctor. Traditional medicine is seen as more relevant to the African ways of living and, most importantly, it is more accessible. The person-to-neurologist ratio in SSA is up to 5,099,908 persons per neurologist, depending on the region. In contrast, the person-to-TH ratio in the SSA is approximately 1:200 (63). The cost of traditional medicines is not necessarily lower than the more affordable AEDs (e.g., phenobarbital), but a consultation with a TH can be considerably cheaper and is viewed as better value for money (64). A TH spends more time with the patient, counsels the whole family and often accepts non-monetary forms of payment, such as home produce or livestock. It is therefore imperative that the educational programs include THs, who can significantly contribute toward removing the stigma of epilepsy and facilitating treatment. There are also social and cultural beliefs attached to genetics and genetic disease. Many religious African communities instill a sense of acceptance and view genetic testing as interference with “god’s will.” This is not necessarily linked to a level of education, but may stem from a lack of understanding of the choices available (65). Therefore, information and counseling is imperative for patients, families, as well as THs. An additional challenge in SSA with its high morbidity and mortality due to HIV/TB/malaria and migrant labor practices is the phenomenon of “orphan households” (66). The high prevalence of households with single or no biological parents renders genetic testing of family trios impossible, complicating research and diagnostic testing protocols. Biological non-paternity is another issue which must be considered, carrying with it significant ethical implications.

To the best of our knowledge, no genetic testing for epilepsy is presently available in SSA. The available genetic testing is generally limited to monogenic diseases and specific, common pathogenic variants. NGS is not routinely performed, though it may sometimes be outsourced through private laboratories, for those who can afford it, as even medical insurance does not always cover the cost. There is also a need for population-based databases and repositories of genomic variants, for correct variant interpretation in the African context.

Therefore, the question that begs asking is whether there is justification in the setting of such obstacles and limited resources, for apparently elite medicine. In our opinion, the answer is “yes” but implementation requires a political and financial engagement from health authorities. Outsourcing of testing to service providers in the HICs presents an economical solution initially, but does not serve to build skills and capacity in Africa. In a middle-income country like South Africa, creating local capacity can be achieved relatively easily with creative use of available infrastructure and an investment in training and human resources. NGS costs are dropping, and manufacturers are focusing on solutions for better scalability and cost-effective analysis of smaller sample batches within clinically relevant turn-around-times. Service-level agreements with local technical service providers (e.g., sequencing facilities affiliated to universities or commercial companies) are being explored.

## Conclusion and Future Directions

Reducing the epilepsy treatment gap in Africa requires improved access to multidisciplinary care (67). The clinical utility of genetic testing in epilepsy presents a compelling case and an opportunity to bring NGS technology into diagnostic laboratories in Africa. It is time to for practical solutions with tangible outputs:

- Training for healthcare professionals in primary healthcare, to create awareness of genetic epilepsies and key clinical identifiers of patients most likely to benefit from testing.
- Education initiatives addressing the misconceptions and prejudices toward epilepsy, genetic disease, and testing aimed at patients, families, community leaders and THs. Here, genetic counselors have an important role to play. SA is the only African country offering Masters-level training for genetic counselors who struggle to find employment post qualification. There is a need for job creation and increased capacity.
- Establishing referral systems between the THs and medical clinics, facilitating access to AEDs, psychosocial support, and genetic counseling.
- It is crucial that the knowledge gained and resources created through projects such as H3Africa and the AGV are accessible to the diagnostic laboratories.
- Initially, small physician–researcher collaborations are likely to drive epilepsy genetic research in Africa. Genetic testing of over 200 patients with DEE is currently underway at the University of Cape Town, in collaboration with the Northwestern University in Chicago. It is hoped that the project will create a basis for a variant database, give rise to a genetic service for epilepsy, and act as a springboard for more epilepsy research in SA and more broadly on the African continent.

## Ethics Statements

This article forms a part of the justification for the genetic epilepsy research referred to in the text, currently underway at University of Cape Town, in collaboration with Northwestern University in Chicago. The research study was granted ethical approval by the Human Research Ethics Committee of the University of Cape Town, in accordance with the Declaration of Helsinki (HREC REF 232/2015).

## Author Contributions

AE: article design, drafting, collation of information, and critical revision; GC and AP: article design and critical revision; RR: critical revision; SK and CN: contribution of epidemiological information and critical revision; JW: article conception, critical revision, and professional communication.

## Conflict of Interest Statement

The authors declare that the research was conducted in the absence of any commercial or financial relationships that could be construed as a potential conflict of interest. GC is a member of the Scientific Advisory Board for Ambry Genetics.
